# Early response of soil fungal communities to the conversion of monoculture cropland to a temperate agroforestry system

**DOI:** 10.7717/peerj.12236

**Published:** 2021-10-05

**Authors:** Lukas Beule, Petr Karlovsky

**Affiliations:** 1 Julius Kühn Institute (JKI)–Federal Research Centre for Cultivated Plants, Institute for Ecological Chemistry, Plant Analysis and Stored Product Protection, Berlin, Germany; 2 Molecular Phytopathology and Mycotoxin Research, Georg-August Universität Göttingen, Goettingen, Lower Saxony, Germany

**Keywords:** Temperate agroforestry, Alley-cropping, Agroforestry establishment, Soil fungi, Amplicon sequencing, Real-time PCR (qPCR)

## Abstract

**Background:**

Alley-cropping systems in the temperate zone are a type of agroforestry in which rows of fast-growing trees are alternated with rows of annual crops. With numerous environmental benefits, temperate agroforestry is considered a promising alternative to conventional agriculture and soil fungi may play a key in maintaining productivity of these systems. Agroforestry systems that are established for more than 10 years have shown to increase the fungal biomass and impact the composition of soil fungal communities. Investigations of soil fungi in younger temperate agroforestry systems are scarce and the temporal dynamic of these changes is not understood.

**Methods:**

Our study was conducted in a young poplar-based alley cropping and adjacent monoculture cropland system in an Arenosol soil in north-west Germany. We investigated the temporal dynamics of fungal populations after the establishment of agroforestry by collecting soil samples half, one, and one and a half years after conversion of cropland to agroforestry. Samples were collected within the agroforestry tree row, at 1, 7, and 24 m distance from the tree row within the crop row, and in an adjacent conventional monoculture cropland. The biomass of soil fungi, Asco-, and Basidiomycota was determined by real-time PCR. Soil fungal community composition and diversity were obtained from amplicon sequencing.

**Results:**

Differences in the community composition of soil fungi in the tree row and arable land were detected as early as half a year following the conversion of monoculture cropland to agroforestry. In the tree row, soil fungal communities in the plots strongly diverged with the age of the system. The presence of young trees did not affect the biomass of soil fungi.

**Conclusions:**

The composition of soil fungal communities responded rapidly to the integration of trees into arable land through agroforestry, whereas the fungal biomass was not affected during the first one and a half years after planting the trees. Fungal communities under the trees gradually diversified. Adaptation to spatially heterogeneous belowground biomass of the trees and understory vegetation or stochastic phenomena due to limited exchange among fungal populations may account for this effect; long-term monitoring might help unravelling the cause.

## Introduction

Agroforestry is the combination of trees and crops in arable land (tree-based intercropping). Modern agroforestry systems in the temperate zone are alley-cropping systems in which rows of fast-growing trees (such as poplar or willow) are alternated with rows of arable crops. The spatial proximity of trees and crops allows for ecological interactions between the tree and crop components of the agroforestry system (Jose, Gillespie & Pallardy, 2004; Schmidt et al., 2020). While these interactions can be complementary or competitional, the main benefits of agroforestry over conventional agriculture are assumed to be the complementary use of resources (Cannell, Noordwijk & Ong, 1996). The most prominent example of the complementary use of resources in agroforestry systems is the ‘safety-net role’ of tree roots where trees take up leached nutrients from deep soil layers that cannot be accessed by the crops (Allen et al., 2004; Wang et al., 2011). Furthermore, these nutrients, which would otherwise be lost from the system, become available to crops again through tree litter deposition in the crop rows and release of nutrients during litter decomposition. This process of recycling leached nutrients by the trees is called ‘nutrient pumping’ (Isaac & Borden, 2019). Additionally, temperate agroforestry has been shown to increase biodiversity (Udawatta, Rankoth & Jose, 2019), pollination services (Varah et al., 2020) as well as soil fertility and erosion control (Torralba et al., 2016) as compared to conventional agriculture. Therefore, agroforestry has been considered a promising land-use system for environmentally sustainable agriculture (Smith, Pearce & Wolfe, 2013).

Temperate agroforestry practice has been shown to affect both microbial abundance and function in soil (Mungai et al., 2005; Banerjee et al., 2016; Beuschel et al., 2019; Beule et al., 2019, 2020; Beule & Karlovsky, 2021; Beule, Arndt & Karlovsky, 2021). For example, by using phospholipid fatty acid analysis, Lacombe et al. (2009) reported greater abundance of arbuscular mycorrhizal (AM) fungi in two temperate agroforestry systems compared to monoculture croplands. Likewise, species richness of AM fungi was found to increase through agroforestry using terminal restriction fragment length polymorphism analysis of fungal 28S rRNA genes from plant roots (Bainard et al., 2012). Amplicon sequencing of soil fungal communities in Canadian temperate alley-cropping systems, revealed that alpha diversity of fungi was lower in the tree rows as compared to eight m distance from the trees within the arable land (Rivest, Whalen & Rivest, 2020). A study that quantified taxonomical fungal groups using real-time PCR (qPCR) revealed that poplar rows in 10-year old alley-cropping systems increased the abundance of soil fungi, particularly Basidiomycota (Beule et al., 2020). At the same sites, amplicon sequencing of soil fungal communities combined with qPCR assays revealed that biomass ectomycorrhizal fungi were strongly promoted in the tree rows (Beule, Arndt & Karlovsky, 2021). Another study that quantified soil fungi in a 4-year old poplar- or alder-based system using qPCR found no promotion of soil fungi through agroforestry (Clivot et al., 2020). In contrast, in a young alder-maize agroforestry system, active biomass of soil fungi obtained by fluorescein diacetate staining was greater in the tree row than in the crop row as early as 3 years after agroforestry establishment (Seiter, Ingham & William, 1999). The determination of total biomass of soil fungi at the same site and sampling dates using differential interference contrast microscopy revealed less consistent trends but still found an increase in fungal biomass under the trees (Seiter, Ingham & William, 1999). There is sufficient evidence that temperate agroforestry does increase soil fungal abundance and affects the assembly of soil fungal communities; however, it is not understood when these alterations are detectable following the conversion of cropland to agroforestry.

This study aimed to investigate the effect of agroforestry on soil fungal communities within the first one and a half years after conversion of a temperate monoculture cropland to a poplar-based alley cropping system. The early effects of agroforestry on soil fungi were determined by sampling soil half, one, and one and a half years after conversion to agroforestry. In these samples, we quantified selected fungal taxa using qPCR and assessed the fungal community composition and diversity using amplicon sequencing. We determined differences among sampling locations on the community composition using pairwise permutational multivariate analysis of variance (PERMANOVA). Multiple univariate diversity measurements (species diversity, evenness, and richness) were applied to assess expected community shifts from multiple perspectives. We hypothesised that the tree row of the agroforestry system (i) increased soil fungal abundance and diversity and (ii) altered community composition as early as 1 year after conversion of cropland monoculture to agroforestry. Seasonal effects as well as responses to the growth stage of the poplar trees by soil fungal communities were expected as different plant growth stages select for different fungi in the rhizosphere (*e.g*. Cavaglieri, Orlando & Etcheverry, 2009). Furthermore, we expected a community shift towards saprotrophic fungi under the trees, which could enhance decomposition rates of tree litter.

## Materials & methods

### Study site and soil sampling

**Figure 1:**
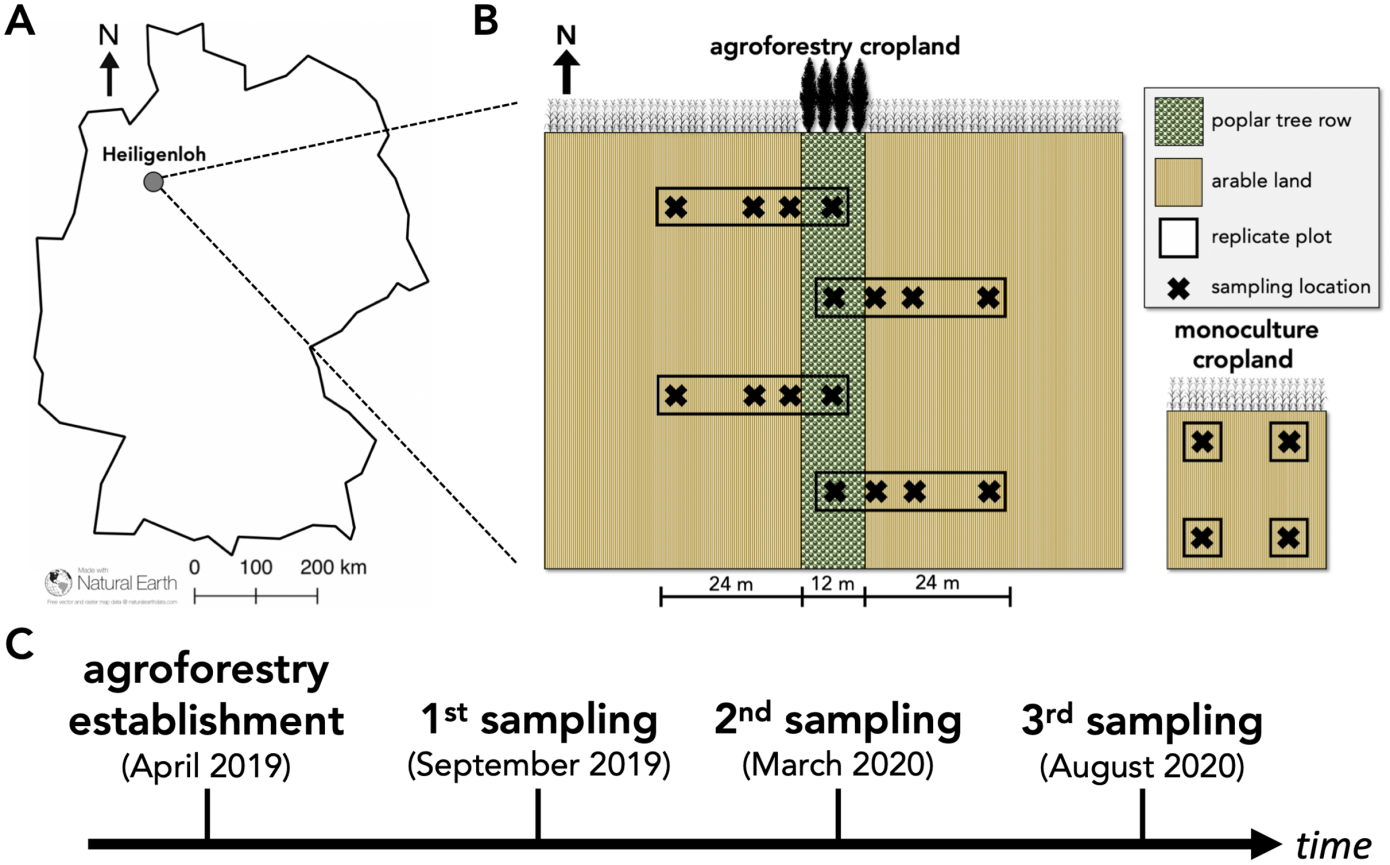
Study site and study design of paired temperate agroforestry and monoculture cropland. Study site in an Arenosol soil near Heiligenloh, Lower Saxony, Germany (A). Soil samples within each replicate plot of the temperate agroforestry cropland system were collected in the centre of the tree row as well as in the crop row at 1, 7, and 24 m distance from the trees. In the monoculture cropland, soil samples were collected in the centre of each replicate plot (B). Timeline of agroforestry establishment and soil sampling (C).

Our study was conducted at an alley-cropping agroforestry system located near Heiligenloh, Lower Saxony, Germany (52°45′29.09″N, 8°32′51.00″E) in an Arenosol soil (Fig. 1A). Adjacent to the agroforestry system, a conventional cropland monoculture served as a reference land use. 1 year prior to the establishment of the agroforestry system, soil properties (bulk density, soil pH, total N, soil organic C, effective cation exchange capacity, base saturation) of the upper 30-cm soil depth were determined by Spanderen (2019) and were comparable among the sampling locations within the yet to be established agroforestry system and adjacent monoculture. The agroforestry crop row was managed identically to the adjacent cropland monoculture (identical crop rotation, fertilization, tillage, and pesticide application). The agroforestry system was established in April 2019 by manually planting a 12-m wide tree row of poplar clones (poplar clone Max 3, *Populus maximowiczii* × *P*. *nigra*) using a dibble bar. As of April 2019, and in accordance with common temperate agroforestry practice (Tsonkova et al., 2012), the agroforestry tree row did not receive fertilizer and was not tilled. The crop rotation of both the agroforestry and monoculture system was maize (*Zea mays*) (2016)–potato (*Solanum tuberosum*) (2017)–winter rye (*Secale cereale*) (2018)–maize (2019)–winter rye (2020). During our sampling years (2019–2020), fertilization rates were 153-73-62 kg N-P-K ha^−1^ yr^−1^ in 2019 and 180-30-131 kg N-P-K ha^−1^ yr^−1^ in 2020.

To capture potential spatial heterogeneities induced by the tree row (*e.g*. through the distribution of tree litter), soil samples within the agroforestry system were collected along transects spanning from the tree row into the crop row (Fig. 1B). Since 48 m is a common width of crop rows in temperate agroforestry systems (*e.g*. Schmidt et al., 2020), soil samples within the agroforestry system were collected in the centre of the tree row as well as at 1, 7, and 24 m (centre of a 48-m wide crop row) distance from the trees within the crop row (Fig. 1B). Soil samples were collected half a year after the establishment of the agroforestry system (September 2019; harvest 2019 prior to tillage), one year after the establishment (March 2020; spring 2020 prior to fertilization), and one and a half years after the establishment (August 2020; harvest 2020 prior to tillage) (Fig. 1C) to investigate temporal effects of agroforestry establishment on soil fungal communities. At each sampling date, a total of 16 soil samples of the upper five cm soil depth were collected in the agroforestry system (*n* = 4) per sampling location (tree row, 1, 7, and 24 m crop row) and four in the adjacent monoculture (*n* = 4) (Fig. 1B). For each soil sample, a total of 750 cm^3^ soil were collected. The samples were thoroughly homogenized and an aliquot of approximately 25 g fresh soil was frozen in the field. Upon arrival at the laboratory, frozen soil samples were freeze-dried for 72 h.

### DNA extraction and real-time PCR

Freeze-dried material was finely ground using a swingmill (MM400, Retsch, Haan, Retsch GmbH) for 60 s at 25 Hz. DNA was extracted from 50 mg soil using a CTAB-based protocol optimized for soils (Beule et al., 2017). Gel electrophoresis was carried out at 4.6 V/cm for 60 min. DNA extracts were tested for PCR inhibitors as described previously (Guerra et al., 2020). Soil extracts were diluted 1:50 (v/v) in double-distilled H_2_O (ddH_2_O) prior to PCR.

Total fungal biomass as well as biomass of Asco- and Basidiomycota in soil samples was estimated using qPCR. Amplification was performed in four µL reaction volume in a CFX384 Thermocycler (Bio-Rad, Rüdigheim, Germany). The composition of the mastermixes and the thermocycling conditions correspond to those described by Beule et al. (2020).

### Amplicon sequencing

Amplification for sequencing library preparation was carried out in 25 µL reaction volume in a peqSTAR 96 thermocycler (PEQLAB, Erlangen, Germany). The reaction volume contained ddH_2_O, buffer (10 mM Tris-HCl, 50 mM KCl, 2.0 mM MgCl_2_, pH 8.3), 200 µM of each deoxynucleoside triphosphate (Bioline, Luckenwalde, Germany), 0.4 µM of each primer (fITS7 (Ihrmark et al., 2012)/ITS4 (White et al., 1990)), one mg mL^−1^ bovine serum albumin, 0.03 u µL^−1^ Hot Start *Taq* DNA Polymerase (New England Biolabs, Beverly, MA, USA), and 6.25 µL of template DNA diluted 1:50 (v/v) in ddH_2_O or ddH_2_O for negative controls. The primers were dual-indexed and included 0–3 frameshifting bases (Ns) to improve base-calling during Illumina sequencing followed by an 8-bp barcode sequence at the 5′-end of each primer to allow multiplexing. Thermocycling conditions consisted of three touch-up cycles (95 °C for 20 s, 50 °C for 30 s, 68 °C for 60 s) followed by 35 cycles of 95 °C for 20 s, 58 °C for 30 s, and 68 °C for 60 s. Final elongation was performed at 68 °C for 5 min. Following amplification, two µL aliquots of the PCR products were visualized on 1.7% (w/v) agarose gels and PCR products were normalized for multiplexing by gel densitometry using ImageJ (Schneider, Rasband & Eliceiri, 2012) as described previously (Beule & Karlovsky, 2021). Adaptor ligation was performed using a commercial kit (Ovation^®^ Rapid DR Multiplex System 1–96) (NuGEN, San Carlos, CA, USA) and amplicons were sequenced using the Illuma MiSeq platform (2 × 300 bp; V3 chemistry) at the facilities of LGC Genomics, Berlin. Sequencing data have been deposited at NCBI’s Sequence Read Achieve (BioProject PRJNA715353).

### Processing of amplicon sequencing data

Sequences were imported in QIIME 2 version 2020.11 (Bolyen et al., 2019) and sequence quality was checked using the ‘q2-demux’ plugin. Forward and reverse sequences were truncated to 200 bp, quality filtered (allowing two expected errors), merged, and chimeras and singletons were removed employing DADA2 (‘q2-dada2’ plugin) (Callahan et al., 2016). The obtained 1,588,263 merged sequences were collapsed into 2,506 exact amplicon sequence variants (ASVs) (Callahan, McMurdie & Holmes, 2017). ASVs were matched against the UNITE database version 8.2 QIIME developer release (Abarenkov et al., 2020) using a scikit-learn naive Bayes machine-learning classifier (‘q2-fit-classifier-naive-bayes’ and ‘q2-classify-sklearn’ plugin) (Pedregosa et al., 2011) in the ‘precision’ configuration to maximize classification precision as suggested previously (Bokulich et al., 2018). After removal of non-fungal ASVs, 1,521,061 sequence counts that clustered into 1,599 ASVs remained. The obtained ASV table was normalized to 1,069 sequence counts per sample using scaling with ranked subsampling (SRS) (Beule & Karlovsky, 2020) employing the ‘SRS’-function in the ‘SRS’ R-package version 2.1.0 (Beule & Karlovsky, 2020) in the R environment version 4.0.3 (R Core Team, 2017).

### Statistical analysis

Each parameter was tested for homoscedasticity (Levene’s test) and normal distribution of the residuals (Shapiro–Wilk’s test). Alpha diversity metrics (Shannon index (*H’*), Chao1 index, and Pielou’s evenness (*J’*)) were calculated from ASV count data employing the ‘vegan’ R package version 2.5–6 (Oksanen et al., 2019). The effect of sampling location (tree row, 1, 7, and 24 m distance from the tree row within the crop row and monoculture cropland) on alpha diversity as well as absolute abundance of fungal groups obtained by qPCR was determined using one-way ANOVA with Tukey’s HSD (for parametric data) or Kruskal–Wallis test with Dunn’s *post hoc* test (for non-parametric data) at *p* < 0.05. ASV count data were square root transformed and the Bray-Curtis index of dissimilarity was calculated pairwise employing the ‘vegdist’-function in the ‘vegan’ R package version 2.5–6 (Oksanen et al., 2019). On the same data, PERMANOVA was performed with 999 permutations (‘adonis2’-function in the ‘vegan’ R package version 2.5–6 (Oksanen et al., 2019)) to test the effect of sampling date, sampling location, and sampling date × sampling location on the composition of the soil fungal community. Pairwise PERMANOVA (‘pairwise.perm.manova’-function in the ‘RVAideMemoire’ R package version 0.9–75 (Hervé, 2020)) with *p*-value correction for multiple comparison using the Benjamini–Hochberg method (Benjamini & Hochberg, 1995) was performed to determine differences among sampling locations on the community composition. The dispersion of samples of the same sampling location at the same sampling date (intra-group dispersion) was measured as the distance from their centroid using multivariate homogeneity of group dispersions on Bray–Curtis dissimilarities (‘betadisper’-function in the ‘vegan’ R package version 2.5–6 (Oksanen et al., 2019)). Soil fungi were classified in saprotrophic and symbiotrophic fungi using the FUNGuild database (Nguyen et al., 2016). Differences in intra-group dispersion and trophic mode among sampling locations at each sampling date were tested using one-way ANOVA with Tukey’s HSD or Kruskal–Wallis test with Dunn’s *post hoc* test at *p* < 0.05. Finally, UpSet plots (‘upset’-function in the ‘UpSetR’ R package version 1.4.0 (Gehlenborg, 2019)) for each sampling location across sampling dates were constructed.

## Results

### Abundance and diversity of soil fungi

**Figure 2:**
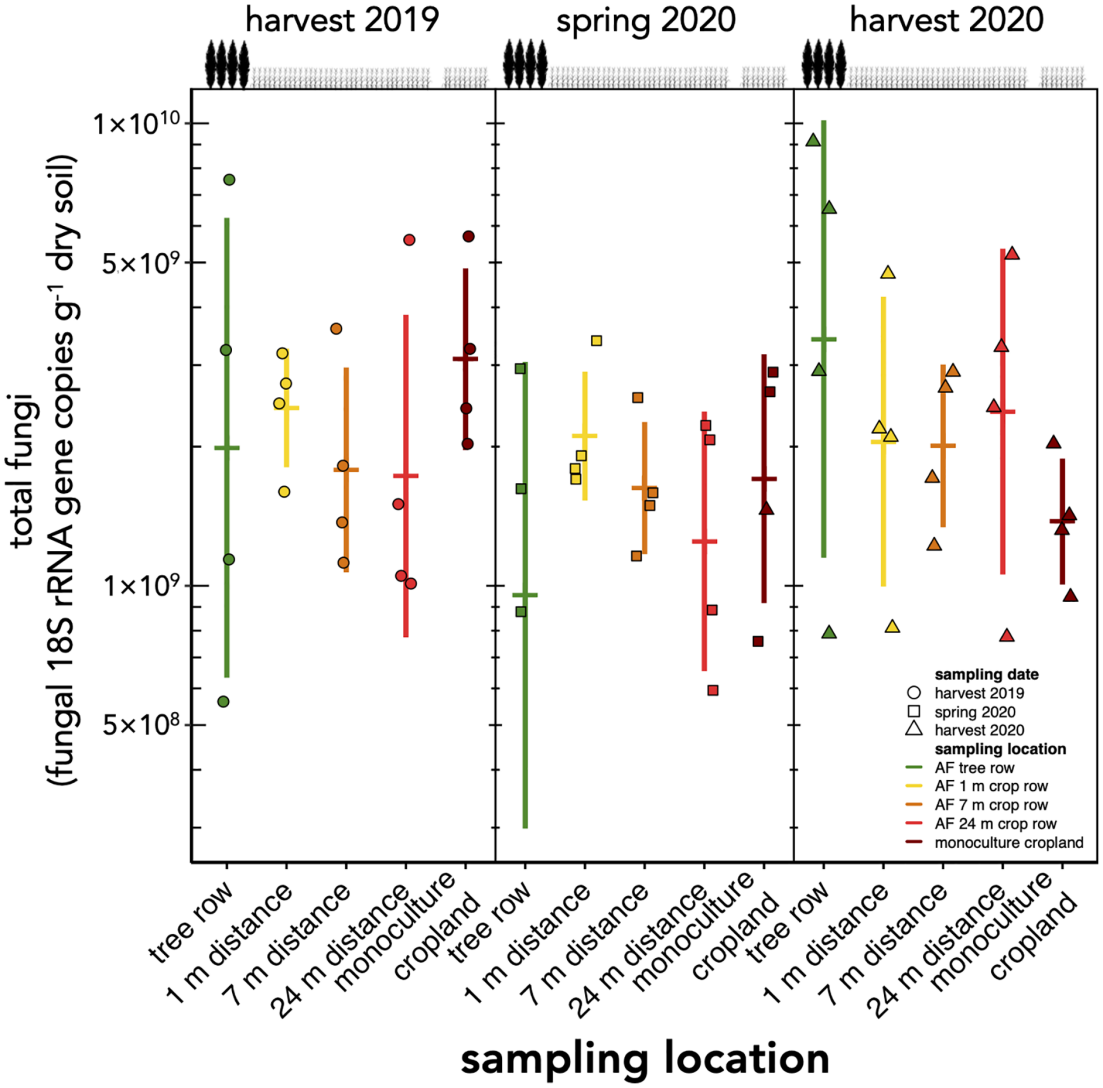
Absolute abundance of soil fungi in a paired temperate agroforestry and monoculture cropland system. Absolute abundances of 18S rRNA genes of all fungi were obtained using real-time PCR. Horizontal bars represent the means, vertical bars the standard deviations (*n* = 4). Circles, squares and triangles represent individual data points collected at harvest 2019, spring 2020, and harvest 2020, respectively. AF = agroforestry system.

The absolute abundance of total soil fungi as well as Ascomycota have not changed within the first one and a half years after the establishment of the agroforestry system (*p* ≥ 0.34) (Fig. 2, Fig. S1). Basidiomycota were detected above the limit of quantification in 28 out of 60 samples and, thus, not analysed statistically.

**Figure 3:**
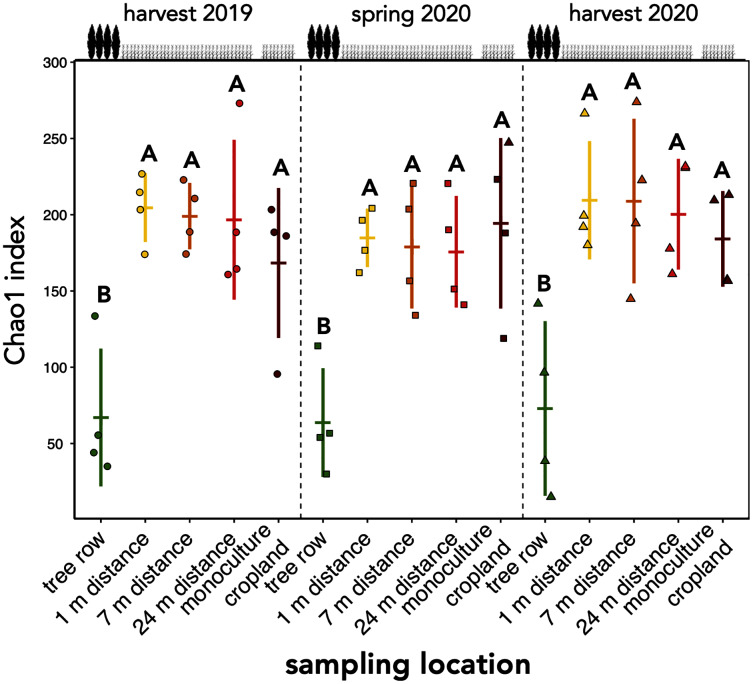
ASV richness of soil fungal communities in a paired temperate agroforestry and monoculture cropland system. Chao1 richness estimate of fungal amplicon sequence variants (ASVs) is shown. Horizontal bars represent the means, vertical bars the standard deviations (*n* = 4). Circles, squares and triangles represent individual data points collected at harvest 2019, spring 2020, and harvest 2020, respectively. Different uppercase letters at the same sampling date indicate significant differences among the sampling locations (tree row, 1, 7, and 24 m distance from the tree row within the crop row of the agroforestry systems, and monoculture cropland) (one-way ANOVA with Tukey’s HSD or Kruskal–Wallis test with Dunn’s *post hoc* test at *p* < 0.05).

The richness of fungal ASVs (measured as Chao1 index) showed a consistent pattern of lower ASV richness in the tree row than in the crop row and monoculture cropland at each sampling date (*p* ≤ 0.02) (Fig. 3). Fungal ASV diversity (measured as Shannon index (*H’*)) was not affected by sampling location at any sampling date (*p* ≥ 0.15) (Fig. S2A). Likewise, no differences among sampling locations were obtained for Pielou’s evenness (*J’*) (Fig. S2B).

### Composition of soil fungal communities

**Figure 4:**
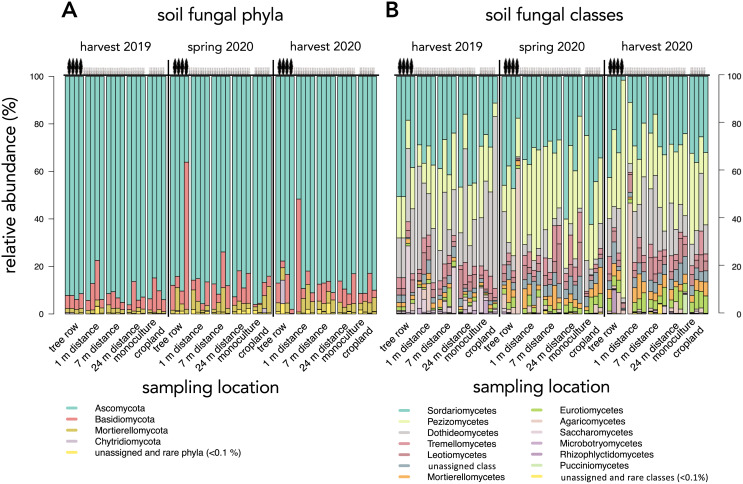
Relative abundance of dominant (≥0.1%) soil fungal phyla (A) and classes (B) in a paired temperate agroforestry and monoculture cropland system. Each stacked bar represents an individual sample.

Across all sampling dates and sampling locations, Ascomycota (87.3 ± 9.6%), followed by Basidiomycota (8.3 ± 9.6%) and Mortierellomycota (3.3 ± 2.5%) were the most dominant phyla (Fig. 4A). Fungal classes were dominated by Sordariomycetes (31.3 ± 11.3%), Pezizomycetes (26.9 ± 13.1%), and Dothideomycetes (16.5 ± 13.8%) (Fig. 4B). *Cladosporium* (9.4 ± 12.0%), *Cephaliophora* (7.6 ± 7.0%), and *Pseudaleuria* (5.3 ± 4.4%) were the most abundant genera and were all found in the phylum of Ascomycota.

**Figure 5:**
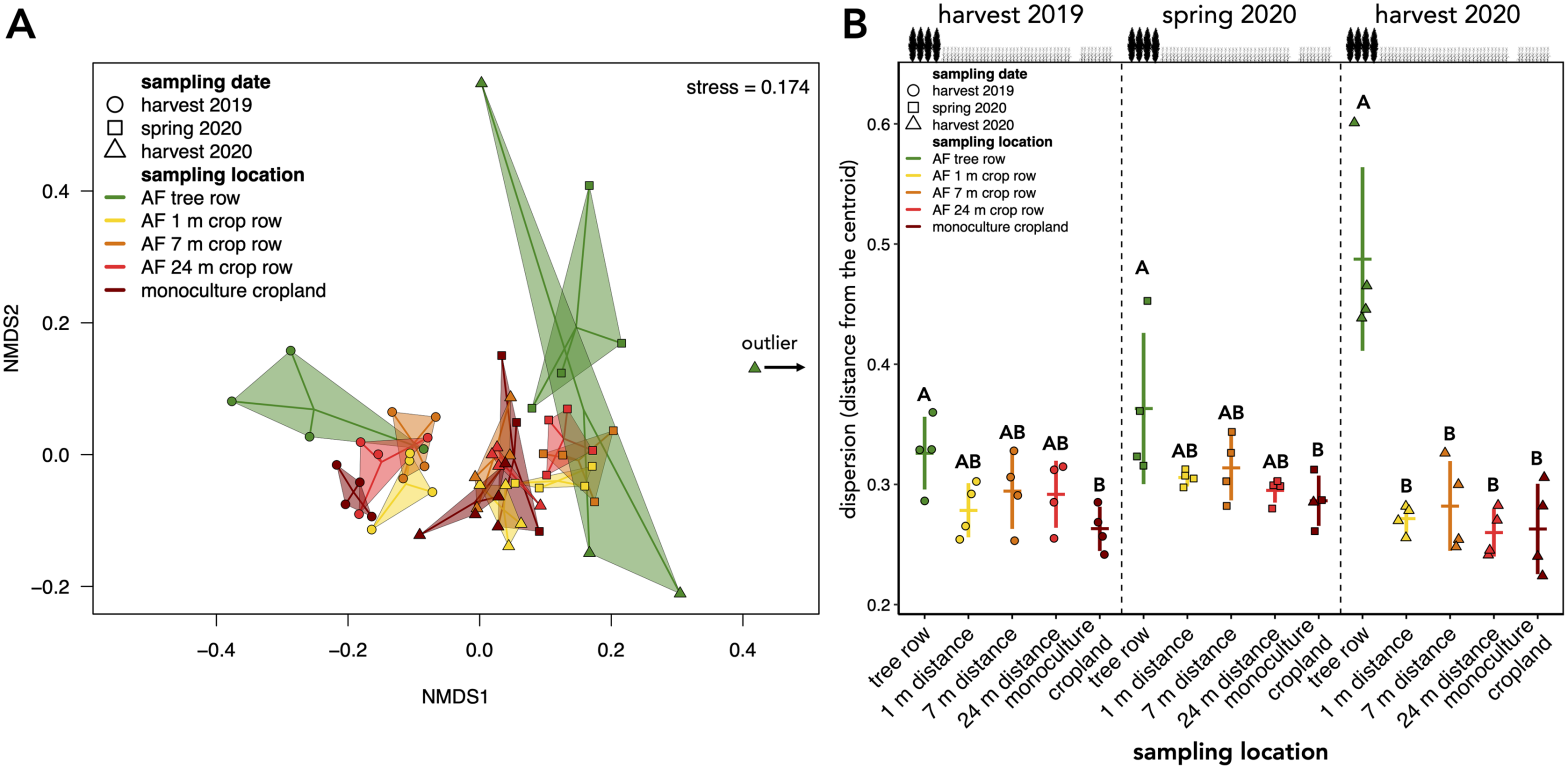
Beta diversity and intra-group dispersion of soil fungal communities in a paired temperate agroforestry and monoculture cropland system. Non-metric multidimensional scaling (NMDS) for pairwise Bray–Curtis dissimilarities is shown; the samples from each location and sampling date are connected with their centroids (A). One sample collected at harvest 2020 was classified as an outlier and is, thus, not shown in (A). Dispersion of samples within each group (sampling locations at each sampling date) is shown in (B). Intra-group dispersion was measured as the distance from the centroid using the analysis multivariate homogeneity of group dispersions (‘betadisper’-function in the ‘vegan’ R-package version 2.5–6 (Oksanen et al., 2019)) on Bray-Curtis dissimilarities is shown. Horizontal bars represent the means, vertical bars the standard deviations (*n* = 4). Different uppercase letters at the same sampling date indicate significant differences among the sampling locations (tree row, 1, 7, and 24 m distance from the tree row within the crop row of the agroforestry systems, and monoculture cropland) (one-way ANOVA with Tukey’s HSD or Kruskal–Wallis test with Dunn’s *post hoc* test at *p* < 0.05). Circles, squares and triangles represent individual samples (*n* = 4) collected at harvest 2019, spring 2020, and harvest 2020, respectively (A, B). AF = agroforestry system.

The community composition of soil fungi was affected by both sampling date and location (Table 1). Pairwise PERMANOVA with the sampling date across sampling locations as a grouping factor confirmed differences in the fungal community composition among sampling dates (*F* = 2.708 to 9.251, *p* = 0.001). These results were reflected by the clustering of the samples in the NMDS ordination, which indicated a temporal dependency of the community composition (Fig. 5A). Apart from the sampling date, sampling location influenced the assembly of the fungal community (*F* = 2.620, *p* = 0.001, Table 1). The clustering in the NMDS ordination suggested that at each sampling date, the agroforestry tree row differed from the agroforestry crop row and monoculture cropland (Fig. 5A), which was confirmed by pairwise PERMANOVA with sampling locations as grouping factor for each sampling date (*F* = 2.178 to 3.042, *p* ≤ 0.006) but different sampling locations within the crop row of the agroforestry system could not be distinguished. The interaction of space and time (sampling date × sampling location) did not affect the community composition (Table 1).

**Table 1:** Permutational multivariate analysis of variance (PERMANOVA) results.

Source of variance	df	Sum Sq	*R* ^*2*^	*F*	*p*-value
Sampling date^a^	2	1.775	0.177	6.862	0.001
Sampling location^b^	4	1.355	0.135	2.620	0.001
Sampling date^a^ × Sampling location^b^	8	1.106	0.110	1.069	0.234
Residuals	45	5.819	0.579		
Total	59	10.055	1.000		

**Notes:**

a Three sampling dates (harvest 2019, spring 2020, harvest 2020).

b Five sampling locations of which four were located in the agroforestry cropland (tree row, 1, 7, and 24 m distance from the tree row) and one in the adjacent monoculture cropland (Fig. 1B).

PERMANOVA was performed with 999 permutations using ASV count data. df = degrees of freedom; Sum Sq = sum of squares; *R*^*2*^ = coefficient of determination; *F* = pseudo − *F* ratio; *p*-values marked in bold indicate statistical significance at *p* < 0.05.

The intra-group dispersion of samples collected in the agroforestry tree row was greater than in the monoculture cropland at harvest 2019 and in the spring 2020 (*p* ≤ 0.031) (Fig. 5B). At harvest 2020, the intra-group dispersion was greater in the tree row as compared to all distances from the tree row within the crop row as well as the monoculture cropland (*p* ≤ 0.0001) (Fig. 5B). Furthermore, the intra-group dispersion within the tree row constantly increased with time and was greater at harvest 2020 than at harvest 2019 (*p* = 0.010) and spring 2020 (*p* = 0.039) (Fig. 5B).

**Figure 6:**
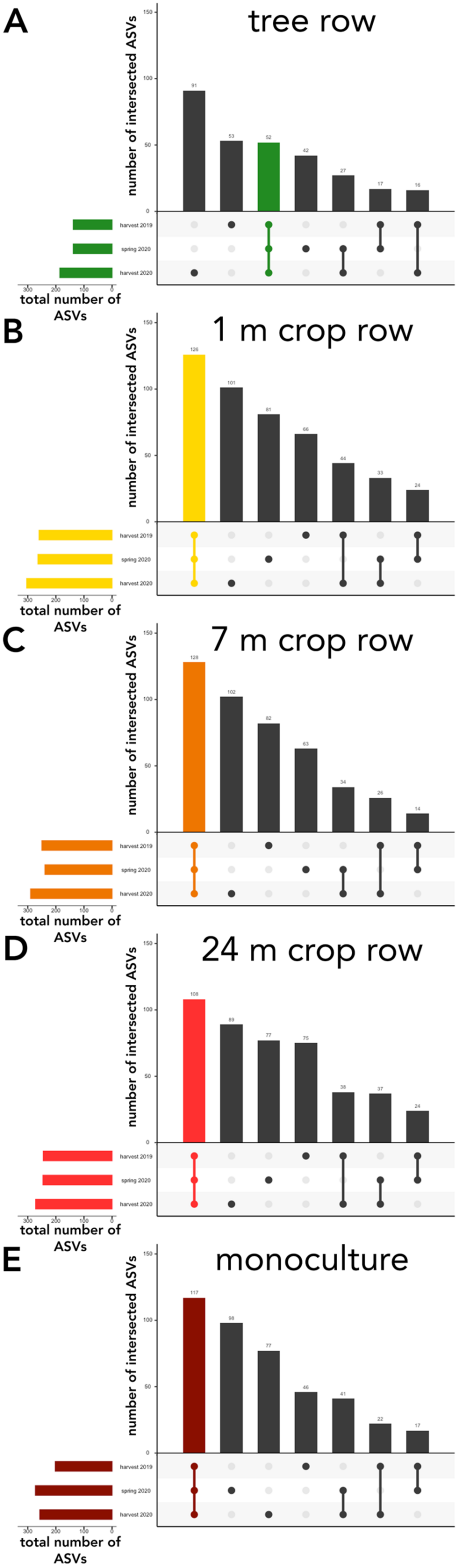
UpSet plot of intersected soil fungal amplicon sequences variants (ASVs) in a paired temperate agroforestry and monoculture cropland system. Soil samples within the agroforestry system were collected in the tree row (A) as well as at 1 (B), 7 (C), and 24 (D) distance from the trees within the crop rows and in an adjacent monoculture cropland (E). Samples in the both management systems were collected at harvest 2021, spring 2020, and harvest 2020.

The sampling locations within the arable land (1, 7, and 24 m within the crop and the monocultures) featured consistently more shared ASVs across all sampling dates than ASVs unique to a specific sampling date (Fig. 6B–6E). In the tree rows, however, the number of ASVs unique to harvest 2019 and 2020 outnumber the ASVs shared across all sampling dates (Fig. 6A).

The relative abundance of saprotrophic fungi was not impacted within the first one and a half years after the establishment of the agroforestry system (Fig. S3A). However, an increase in symbiotrophic fungi in tree row was noted after one and a half years (Fig. S3B). Most of these symbiotrophic fungi were ectomycorrhizal fungi.

## Discussion

Our results demonstrated that differences in the community composition and richness of soil fungi in the tree row and arable land (crop row and adjacent monoculture cropland) were detected as early as half a year following the conversion of monoculture cropland to agroforestry. Furthermore, we found that soil fungal communities in plots of the tree row strongly diverged with the age of the systems and undergo a faster community relative to those in the arable land.

Contrary to our expectation, but in agreement with the findings obtained in a 4-year old agroforestry system in France (Clivot et al., 2020), the tree row of our agroforestry system did not increase fungal abundance within the first one and a half years following agroforestry establishment (Fig. 2). The work of Seiter, Ingham & William (1999) may explain these findings. In their study, the active fungal biomass was greater in the tree row than in the crop row as early as 3 years after agroforestry establishment, whereas the total fungal biomass showed fewer differences between trees and crops. Furthermore, the active fungal biomass accounted just for a small proportion of the total fungal biomass (Seiter, Ingham & William, 1999). Since the present study as well as the work of Clivot et al. (2020) did not distinguish among active, dormant or dead fungal biomass, the response of the fungal biomass to agroforestry may have been restricted to the active biomass and, thus, may have remained undetected in our study.

In contrast to the study sites of Beule et al. (2020), who found strong promotion of soil fungi through 10-year old poplar tree rows, the soil under our young tree row did not receive long-lasting tree litter input that could serve as a growth substrate for litter-decomposing fungi such as members of the phylum Basidiomycota (Lundell et al., 2014). We assume that the stimulation of soil fungi in the tree row as compared to cropland due to the absence of tillage (Frey, Elliott & Paustian, 1999; Mathew et al., 2012) would need a longer time to manifest than the age of our agroforestry system allowed.

In older temperate agroforestry systems, a permanent herbaceous understory vegetation has frequently been reported under the trees (Battie-Laclau et al., 2019; Boinot et al., 2019; Beule et al., 2020; D’hervilly et al., 2020). Plants growing under the trees preserve plant diversity in the system. This self-evident fact has recently been reported (Boinot et al., 2019). Although the biomass of the understory vegetation is negligible as compared to the trees’, the decomposition of litter from diverse herbaceous plants with a high diversity of secondary metabolites can affect soil microbial communities (Chomel et al., 2016). Furthermore, the importance of understory vegetation for maintaining AM fungi has been reported recently (Battie-Laclau et al., 2019). From our observations during sampling campaigns and maintenance of the study site, we recognized that the spontaneous understory vegetation of the tree row was poorly developed during the study period. We noticed sporadic occurrence of Canadian horseweed (*Conyza canadensis*) and annual grasses. Since overstory age affects the species composition of the understory vegetation (*e.g*. Whitney & Foster, 1988), the understory vegetation is likely to change with increasing tree age. Based on our previous investigation of older agroforestry systems (Beule et al., 2020), we assume that with increasing age of the agroforestry system, the tree row will develop into a habitat that harbours more fungal biomass than the surrounding arable land.

Poplar trees affected the community composition of soil fungi no later than half a year after the conversion of conventional monoculture cropland to agroforestry (Fig. 5A), confirming our second hypothesis. On a temporal scale, our results revealed an overall fast community turnover, which is greatest within the tree rows (Fig. 6), implying that young poplar trees rapidly shift soil fungal community composition as they develop. This supports our hypothesis that the establishment of tree rows in arable land through agroforestry has immediate impact on soil microbial community composition. Furthermore, this agrees with our expectation that not just different plants but also different plant growth stages select fungi in the rhizosphere. However, besides rhizosphere effects, it has to be noted that tree rows affect microclimatic conditions (Kanzler et al., 2018), which could explain seasonal effects on community composition.

Our findings agree with previous studies that showed that tree rows in temperate agroforestry systems altered microbial communities in soil (Mungai et al., 2005; Bainard et al., 2011, 2012; Beuschel et al., 2019; Beule et al., 2019, 2020; Beule & Karlovsky, 2021; Beule, Arndt & Karlovsky, 2021). The reduction of fungal richness (Fig. 3) and the stagnation of fungal biomass under the trees (Fig. 2) as compared to the crop row and monoculture at every sampling date indicated that fewer fungi with a larger share of biomass were present in the tree row. Over time, the input of substantial amounts of tree litter in the poplar row (Swieter et al., 2018) is expected to favour the growth of specific fungal decomposers (Urbanová, Šnajdr & Baldrian, 2015), the composition of which shifts with the state of decomposition (Voříšková & Baldrian, 2013). In our young agroforestry system, however, we did not identify an increased abundance of saprotrophic fungi (Fig. S3A), indicating that our system is too young to for the establishment of a substantial decomposer community. Furthermore, differences in the amount and composition of root exudates between the poplar trees and the arable crops can shape soil fungal communities (Broeckling et al., 2008). Therefore, we assume that differences in plant-derived nutrients (tree litter and root exudates) between the tree row and arable land contributed to the observed differences the composition of the soil fungal community between tree rows and cropland. Positive effect of the absence of tillage on fungal communities in the tree row has likely not been observed due to the young age of the system (see above). Therefore, in addition to the input of plant-derived nutrients, differences in management practice between the tree row and the arable land likely contributed to the differences in the community composition.

We observed continuous diversification of soil fungal communities in the tree row with increasing agroforestry age (Fig. 5B). This unexpected finding can be accounted for by adaption of soil fungi heterogeneous environments under the tree rows or by stochastic processes in subpopulations with limited exchange of microflora. We have no evidence for the heterogeneity of the aboveground tree litter deposition since the poplar trees were clones planted at fixed distances from each other, and spontaneous growth of other tree species was not observed. The lateral and vertical distribution of poplar roots was not determined; however, considerable variation in total root mass and length of poplar clones of young age (1 to 3 years) has been reported in the field (Douglas, McIvor & Lloyd-West, 2016). Combined with the high spatial heterogeneity of the spontaneous herbaceous understory vegetation, spatial differences in belowground litter and root exudate inputs may have accounted for the observed divergence of soil fungi under the trees. Alternatively, low fungal species richness under the trees following the conversion of arable land to agroforestry (Fig. 3) and limited exchange of microflora among sampling locations may have reinforced stochastic processes, leading to random diversification of the communities in the tree rows (Ramoneda et al., 2020; Grilli, 2020). As compared to the arable land, the absence of tillage in the tree rows likely contributed to mutual isolation of fungal populations in soil. Interestingly, the interaction of space and time did not affect fungal community composition (Table 1). The absence of space-time interaction may serve as an indicator that stochastic rather than deterministic processes control the assembly of microbial communities in soil. Both hypotheses, adaptation to belowground heterogeneities and stochastic community assembly, imply that the differences among populations will decline with age of the tree row.

## Conclusion

The integration of poplar trees into arable land through temperate alley cropping altered the community composition of soil fungi in the tree row as compared to the arable land as early as half a year following the conversion of monoculture cropland to agroforestry. Tree rows reduced fungal richness but gradually increased community dispersion with age of the agroforestry system, which is expected to yield in large community changes in the long term. Although fungal biomass, evenness, and diversity were not impacted, community composition was highly dynamic in time, pointing to the importance of (i) community composition metrics rather than univariate diversity metrics and (ii) longitudinal investigations of soil microbiomes. Short-term monitoring did not allow us to distinguish between two potential causes of the diversification: (i) spatial heterogeneity of belowground biomass of the trees and understory vegetation, and (ii) stochastic community assembly due to the lack of exchange among fungal populations in soil under the trees.

## Supplemental Information

10.7717/peerj.12236/supp-1 Supplemental Information 1 Absolute abundance of Ascomycota in soil of a paired temperate agroforestry and monoculture cropland system. Absolute abundances of internal transcribed spacer (ITS) genes of all Ascomyycota were obtained using real-time PCR. Horizontal bars represent the means, vertical bars the standard deviations (*n* = 4). Circles, squares and triangles represent individual data points collected at harvest 2019, spring 2020, and harvest 2020, respectively. AF = agroforestry system.

10.7717/peerj.12236/supp-2 Supplemental Information 2 Shannon diversity index (*H’*) (A) and Pielou’s evenness (*J’*) (B) of amplicon sequence variants (ASVs) in a paired temperate agroforestry and monoculture cropland system. Horizontal bars represent the means, vertical bars the standard deviations (*n* = 4). Circles, squares and triangles represent individual data points collected at harvest 2019, spring 2020, and harvest 2020, respectively. AF = agroforestry system.

10.7717/peerj.12236/supp-3 Supplemental Information 3 Relative abundance of saprotrophic (A) and symbiotrophic fungi (B) in a paired temperate agroforestry and monoculture cropland system. Fungal amplicon sequencing variants (ASVs) were classified using FUNGuild. Horizontal bars represent the means, vertical bars the standard deviation (*n* = 4). Circles, squares, and triangles represent individual data points collected at harvest 2019, spring 2020, and harvest 2020, respectively. AF = agroforestry system.

## Additional Information and Declarations

### Field Study Permissions

The following information was supplied relating to field study approvals (*i.e*., approving body and any reference numbers): Soil sampling was conducted on a privately-owned land by G. Lampe & Dr. J. Langenberg (Agrar Lampe Services, Germany) who gave us access to the study site and allowed us to sample soil.

### Data Availability

The following information was supplied regarding data availability: The sequencing data is available at NCBI SRA: PRJNA715353.
