# Supplementary figures and images for: Early response of soil fungal communities to the conversion of monoculture cropland to a temperate agroforestry system
Source: PeerJ. 2021 Oct 5;9:e12236. doi: 10.7717/peerj.12236 (PMC8500075; doi:10.7717/peerj.12236)

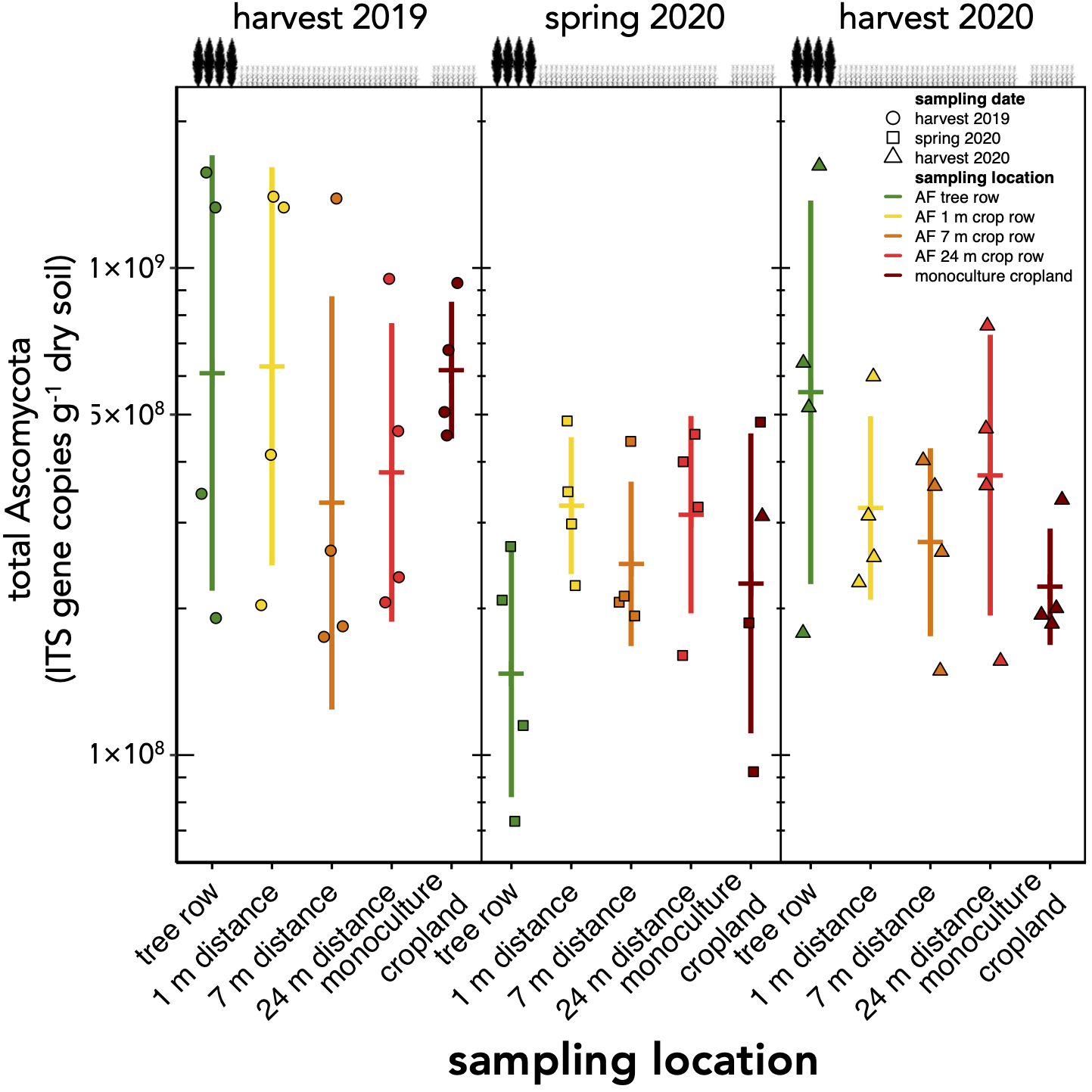

Supplement: Supplemental Information 1 — Absolute abundances of internal transcribed spacer (ITS) genes of all Ascomyycota were obtained using real-time PCR. Horizontal bars represent the means, vertical bars the standard deviations (n = 4). Circles, squares and triangles represent individual data points collected at harvest 2019, spring 2020, and harvest 2020, respectively. AF = agroforestry system. [file peerj-09-12236-s001.png]

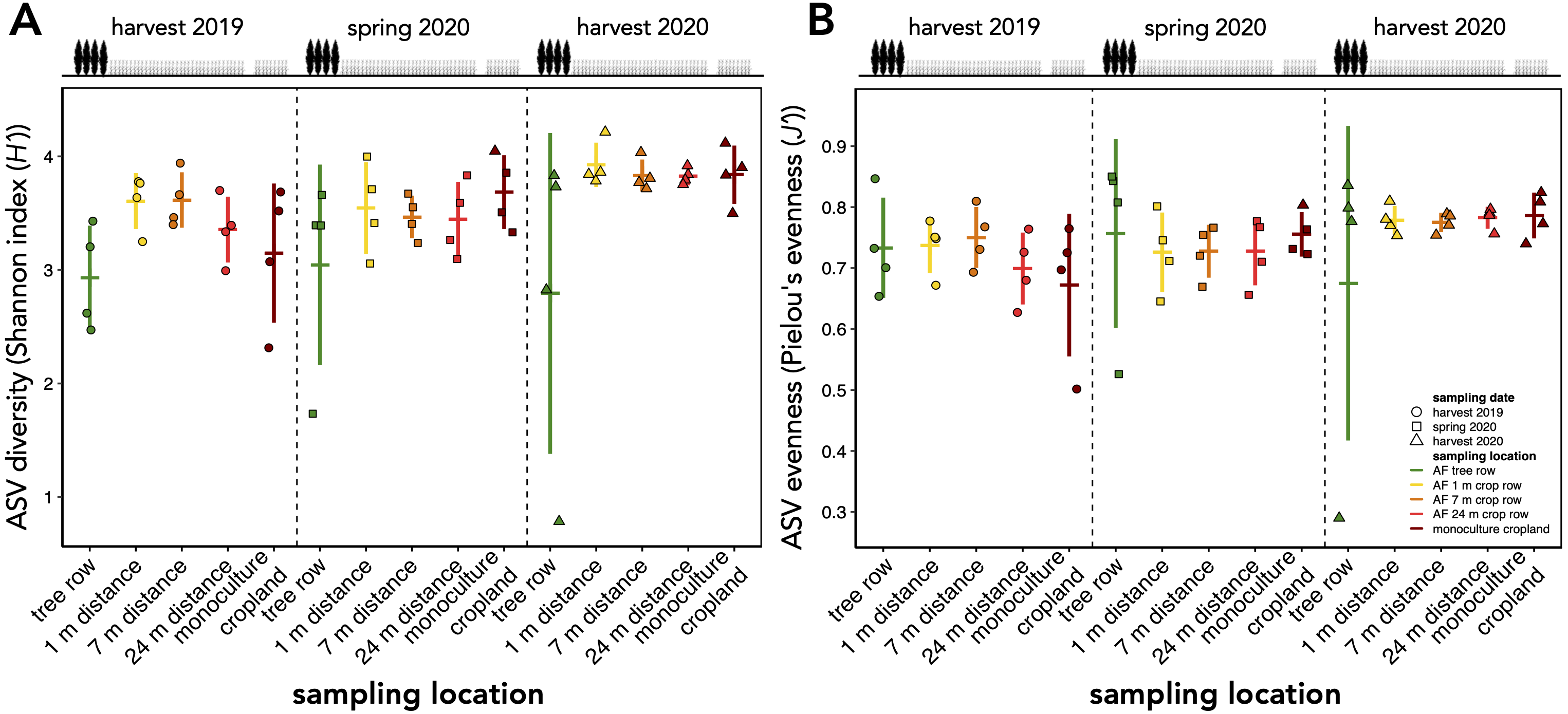

Supplement: Supplemental Information 2 — Horizontal bars represent the means, vertical bars the standard deviations (n = 4). Circles, squares and triangles represent individual data points collected at harvest 2019, spring 2020, and harvest 2020, respectively. AF = agroforestry system. [file peerj-09-12236-s002.png]

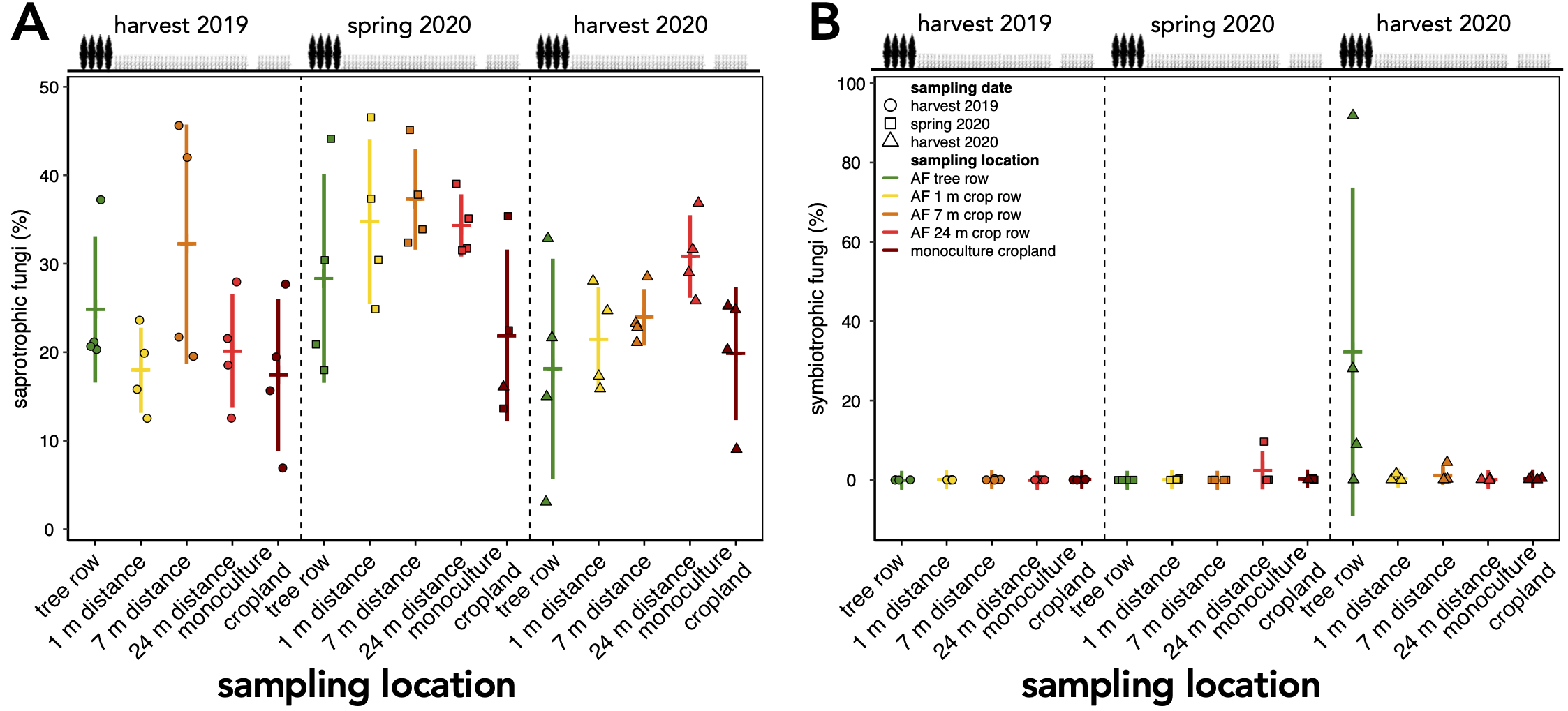

Supplement: Supplemental Information 3 — Fungal amplicon sequencing variants (ASVs) were classified using FUNGuild. Horizontal bars represent the means, vertical bars the standard deviation (n = 4). Circles, squares, and triangles represent individual data points collected at harvest 2019, spring 2020, and harvest 2020, respectively. AF = agroforestry system. [file peerj-09-12236-s003.png]
